# Assessing the effects of maternal HIV infection on pregnancy outcomes using cross-sectional data in Malawi

**DOI:** 10.1186/s12889-020-09046-0

**Published:** 2020-06-22

**Authors:** Halima S. Twabi, Samuel O. Manda, Dylan S. Small

**Affiliations:** 1 Department of Mathematical Sciences, University of Malawi, Zomba, Malawi; 2 Biostatistics Research Unit, South Africa Medical Research Council, Pretoria, South Africa; 3 School of Mathematics, Statistics and Computer Science, University of Kwazulu-Natal, Pietermaritzburg, South Africa; 4 Department of Statistics, University of Pretoria, Pretoria, South Africa; 5 Department of Statistics, University of Pennyslvania, Pennyslvania, USA

**Keywords:** Propensity score, Confounders, Maternal HIV, Inverse weighting, Perinatal mortality, Antiretroviral therapy

## Abstract

**Background:**

Several studies have shown that maternal HIV infection is associated with adverse pregnancy outcomes such as low birth weight and perinatal mortality. However, the association is conflicted with the effect of antiretroviral therapy (ART) on the pregnancy outcomes and it remains unexamined. If the association is confirmed then it would guide policy makers towards more effective prevention of mother to child HIV transmission interventions. Using methods for matching possible confounders, the objectives of the study were to assess the effect of maternal HIV infection on birth weight and perinatal mortality and to investigate the effect of ART on these two pregnancy outcomes in HIV-infected women.

**Methods:**

Data on 4111 and 4759 children, born within five years of the 2010 and 2015-16 Malawi Demographic and Health Surveys (MDHS) respectively, whose mothers had an HIV test result, were analysed. A best balancing method was chosen from a set of covariate balance methods namely, the 1:1 nearest neighbour (NN) matching, matching on the propensity score (PS) and inverse weighting on the PS. HIV and ART data were only available in the MDHS 2010, permitting an assessment of the moderating effect of ART on the association between maternal HIV infection and birth weight and perinatal mortality.

**Results:**

The overall average birth weight was 3227.9g (95% CI: 3206.4, 3249.5) in 2010 and 3226.4g (95%: 3205.6, 3247.2) in 2015-16 and perinatal mortality was 3.8% (95%: 3.2, 4.3) in 2010 and 3.5% (95%: 2.8, 3.8) in 2015-16. The prevalence of HIV among the mothers was 11.1% (95%: 10.1, 12.0) and 9.2% (95% CI: 8.4, 10.1) in 2010 and 2015-16, respectively. In 2010, maternal HIV infection was negatively associated with birth weight (mean= -25.3g, 95% CI:(-95.5, -7.4)) and in 2015-16 it was positively associated with birth weight (mean= 116.3g, 95% CI:(27.8, 204.7)). Perinatal mortality was higher in infants of HIV-infected mothers compared to infants of HIV-uninfected mothers (OR = 1.5, 95% CI:(1.1 - 3.1)) in 2010, while there was no difference in the rate in 2015-16 (OR = 1.0, 95% CI:(0.4, 1.6)). ART was not associated with birth weight, however, it was associated with perinatal mortality (OR=3.9, 95% CI:(1.1, 14.8)).

**Conclusion:**

The study has found that maternal HIV infection had an adverse effect on birth weight and perinatal mortality in 2010. Birth weight was not dependent on ART uptake but perinatal mortality was higher among infants of HIV-infected mothers who were not on ART. The higher birth weight among HIV-infected mothers and similarity in perinatal mortality with HIV-uninfected mothers in 2015-16 may be indicative of successes of interventions within the PMTCT program in Malawi.

## Background

Maternal HIV infection has been shown to be associated with increased rates of adverse pregnancy outcomes such as low birth weight (LBW) (weighing less than 2500g at birth) and increased perinatal and early neonatal mortality [1–8]. The adverse impacts are largely due to maternal and obstetric complications among HIV-infected women [9, 10]. Nearly 20.5 million infants globally were born with low birth weight in the year 2015 [11]. Studies have shown that low birth is associated with high levels of child morbidly and mortality [12]. Infant mortality and morbidity persist in sub-Saharan African (SSA) region in which the HIV prevalence among pregnant women is high [13]. Identifying important and modifiable factors that influence LBW and perinatal mortality in a population with a high HIV burden, improves understanding of the pathways underlying the association between maternal HIV infection and pregnancy outcomes. In addition, it provides supporting scientific evidence for possible prevention and intervention within the Prevention of Mother-to-child HIV transmission (PMTCT) programme.

A growing number of studies have shown conflicting evidence on the association between maternal HIV and adverse pregnancy outcomes such as LBW. Some studies have shown that antiretroviral therapy (ART) among HIV-infected mothers is associated with adverse outcomes such as low birth weight (LBW), intrauterine growth restriction (IUGR), preterm delivery (PTD) and stillbirths [14–18]. On the other hand, several studies have found that sustained intake of ART among HIV-infected mothers reduces adverse pregnancy outcomes such as stillbirths, low birth weight and prematurity [16, 19]. Despite the disparity in the evidence, statistical methods can be used to determine the moderating effect of ART on the association between maternal HIV-infection and birth weight and perinatal mortality.

Most of the evidence on the association between maternal HIV and pregnancy outcomes comes from observational studies. However, establishing the impact of maternal HIV infection on birth weight and perinatal mortality is challenging because there could be other purported factors besides maternal HIV infection associated with pregnancy outcomes. The presence of confounding factors misrepresents the impact of maternal HIV infection on pregnancy outcomes as the distribution of these factors may be different between HIV-infected and HIV-uninfected mothers. Maternal anaemia, genitourinary tract infection in pregnancy, hypertension, history of birth weight and birth order are some of the factors known to be associated with pregnancy outcomes [14–17, 20–22]. Additionally, socio-economic and environmental factors such as maternal education, maternal age, poverty levels, air pollutants and smoking are associated with pregnancy outcomes [22–26]. To ensure comparability between HIV-infected and HIV-uninfected mothers, a randomised control trial (RCT) would ideally need to be conducted. However, since most HIV studies are observational, using methods developed by Rosenbaum and Rubin [27], one could achieve the same task of balancing confounder variables between HIV-infected and HIV-uninfected mothers. For example, one such method is based on Propensity Score (PS) matching, which aims to emulate the randomisation procedure of RCTs, as the distribution of measured confounders between exposure groups is made similar by using the PS [27–33, 33–35].

Ascertaining the association between maternal HIV infection and birth weight and perinatal mortality could be conflicted by the association between ART and the outcomes in HIV-infected mothers. The study therefore aimed at assessing the effect of maternal HIV infection on birth weight and perinatal mortality, and to investigate the effect of ART on the two pregnancy outcomes in HIV-infected women. This was done within an appropriate application of causal inference statistical methods to balance a set of confounder variables. The association between maternal HIV infection and birth weight and perinatal mortality with and without controlling for ART uptake in a high HIV epidemic and ART uptake population, remains largely untested. Confirmation of these associations would help policy makers obtain robust evidence-based findings that would assist in providing effective prevention of mother to child HIV transmission (PMTCT) interventions.

## Methods

### Study data

The data from this study were obtained from the 2010 and 2015-16 Malawi Demographic and Health Surveys (MDHS). These are nationally representative household surveys that provide data for a wide range of population, health, and nutrition indicators in low-income and middle-income (LIMICs). The Demographic and Health Surveys are implemented by the Demographic Health Survey Programme which is funded by the United States Agency for International Development (USAID). The two Malawian surveys provided up-to-date information on current HIV trends, child health and nutrition. Additionally, the 2010 survey provided information on ART. Parents of infants included in the surveys had signed an informed consent. Details of the sampling design for the MDHS can be obtained from the 2010 and 2015-16 reports [36, 37]. The study analyzed data on 4,111 and 4,759 children born to mothers who had an HIV result during the respective surveys. The outcome variables of interest generated were: Birth weight (grams) and perinatal mortality (0= Alive 1= Dead). The exposure variable of interest was mothers’ HIV-infection status (0 = HIV negative, 1 = HIV positive). The moderator variable was ART status among HIV-infected mothers (0 = Not on ART, 1 = On ART) using the 2010 MDHS. The effect estimates were obtained for the effect of maternal HIV infection on birth weight and perinatal mortality.

From the surveys, the overall average birth weight was 3227.9g (95% CI: 3206.4, 3249.5) and 3226.4g (95% CI: 3205.6, 3247.2) in 2010 and 2015-16, respectively. In 2010 the mean birth weight of children born to HIV-infected and HIV-uninfected mothers was 3196.6g (95% CI: 3125.3, 3267.9) and 3231.4g (95% CI: 3208.8, 3254.0), respectively. While in 2015-16, the mean birth weight was 3276.4g (95% CI: 3203.6, 3349.1) for children born to HIV-infected mothers and 3221.4g (95% CI: 3199.7, 3243.1) for children born to HIV-uninfected mothers. Perinatal mortality was recorded as 38 deaths per 1000 live births and 35 deaths per 1000 live births in 2010 and 2015-16, respectively. In 2010, 11.1%(95% CI: 10.1, 12.0) mothers were HIV-infected and 88.9%(95% CI: 88.0,90.0) mothers were HIV-uninfected and in 2015-16, 9.2%(95% CI: 8.4,10.1) mothers were HIV-infected and 91.2%(95% CI: 90.4, 91.9) mothers were HIV-uninfected. Perinatal mortality was recorded as 55 per 1000 live births among HIV-infected mothers and 40 per 1000 live births among HIV-uninfected mothers in 2010. In 2015-16, perinatal mortality was recorded as 37 per 1000 live births among HIV-infected mothers and 31 per 1000 live births among HIV uninfected mothers.

#### Maternal HIV-infection data collection

For both the 2010 and 2015-16 MDHS, interviewers collected finger-prick blood specimens from women age 15-49 years and men age 15-54 years who consented to laboratory HIV testing. The protocol for blood specimen collection and analysis was based on the anonymous linked protocol developed for the DHS Program. This protocol allows for the merger of HIV test results with the socio-demographic data collected in the individual questionnaires after removal of all information that could potentially identify an individual. Interviewers explained the procedure, the confidentiality of the data, and the fact that the test results would not be made available to respondents [36, 37]. A sub-sample of 7924 and 8271 women were tested for HIV using an ELISA test during the 2010 and 2015-16 MDHS, respectively. The data set used in the analysis was children data which was based on woman and household questionnaires.

### Statistical analysis

The maternal HIV infection effect was estimated for birth weight and perinatal mortality by comparing the outcomes *ATE*=*E*[ *Y*_*i*_(1)−*Y*_*i*_(0)] between HIV-infected and HIV-uninfected mothers across covariates that were considered as possible confounders using standard statistical balancing tools. The distribution of the covariates must not differ across the two HIV groups, so that the effect on birth weight or on perinatal mortality should be attributed to HIV infection only, if no other unmeasured confounding is present. Three approaches that balance confounders were assessed and the best method was chosen to estimate the effect of maternal HIV infection on birth weight and perinatal death. A brief description of three matching methods; a combination of exact and nearest neighbour matching, the propensity score matching and the inverse probability weighting on the PS are presented in the following subsections. The balancing performance of the three methods is assessed using the standardised mean difference (SMD). The method that yielded the best balance was then used to assess the association between HIV status on birth weight and perinatal mortality.

#### Identifying confounders from the data

Several variables were identified from the MDHS data as potential confounders for the effect of maternal HIV on birth weight. These include; region, place of residence, wealth, age, maternal anaemia, smoking status of a mother, education level, size of child at birth, anaemia, ever terminated a pregnancy, mothers’ body mass index (BMI) and marital status. For the effect of maternal HIV infection on perinatal mortality, we identified residence, place of delivery, maternal anaemia, size of child at birth, ever terminated pregnancy, body mass index (BMI), education of a mother and wealth as potential confounders. A list of the variables, that significantly affected HIV infection status and the pregnancy outcomes for both the 2010 and 2015-16 data, are shown in Table 8 in the Appendix.

#### Handling missing data

The MDHS data had some missing data on important variables and hence it was essential to control for missing data. We initially did a visualisation of the missing pattern among our variables of interest as displayed in Table 7 in the Appendix. We noted that the data had more than 10% of missing data. Hence, taking into consideration that severity may have been affected by missing data, a multiple imputation on the missing data was done for both the 2010 and 2015-16 MDHS dataset. The mice package in R was used. All further analysis were done on the imputed data.

#### Exact and nearest neighbour (NN) matching

Exact matching was used to match observed covariates between HIV-infected and HIV-uninfected mothers. If we let *i* to represent unmatched HIV-infected mothers and *j* to be a set of unmatched HIV-uninfected mothers, then exact matching ensures that for measured covariates *X*, *X*_*i*_=*X*_*j*_=*x* [31, 38]. The exact matching was combined with the traditional nearest neighbour matching, which considers the distance metrics between covariates, [39] to address the problem of increase in dimensionality that comes with exact matching only on the covariates [40]. The 1:3 nearest neighbour matching was used to select for each HIV-infected mother *i*, three corresponding HIV-uninfected mothers *j* with the smallest distance between them. HIV-infected and HIV-uninfected mothers were matched on the covariates region, residence, anaemia, age, body mass index (BMI), size of child at birth and wealth using the NN matching for both datasets. Exact match was done on covariates region, ever terminated pregnancy and mothers’ age.

#### Matching based on the propensity score (PS)

An alternative to the traditional matching method between HIV-infected and HIV-uninfected on the covariates is known as a propensity score. The propensity score [27, 41] is the probability that a subject is HIV-infected conditional on observed covariates, denoted as;

1 $$ e(x_{i})=P(\text{HIV} = 1 \mid x_{i})  $$

The propensity score was estimated using a multiple binary logistic regression with exposure (HIV status = 1 or 0) as an outcome variable against potential confounders. The propensity score, known as a balancing score, can produce unbiased average treatment effects [27] under a common assumption known as the strong ignorability assumption. The model is as follows;

$${}\begin{aligned} P(\text{HIV Status} &=1 \mid X_{1})= \text{log} \left(\frac{\pi(x)}{1-\pi(x)} \right) \\ &= \beta_{0} + \beta_{1} \text{Region} + \beta_{2} \text{mothers age}\\ &\quad+ \beta_{3} \text{residence} + \beta_{4} \text{Wealth}\\& \quad+ \beta_{5} \text{pregnancy termination}\\& \quad+ \beta_{6} \text{anaemia} + \beta_{7} \text{birth size} + \beta_{8}\text{BMI} \end{aligned} $$

The vector of parameters *β* were estimated based on the whole sample. *π*(*x*) is the probability of a mother being HIV-infected (*Y*=1). Subjects were 1:1 matched without replacement on the logit of the propensity using Austin’s [42] recommended caliper of 0.2, as it is known to produce low mean square error (MSE).

#### Inverse probability weighting (IPW) based on the PS

The inverse probability weight has been known to perform better in controlling confounding unlike matching on the propensity score [34, 38]. The PS weighting re-weights HIV-infected and HIV-uninfected mothers by creating a population that is free from measured bias [28]. The inverse of the probability of a mother being HIV-infected, conditional on covariates in the data (inverse of the PS) is given by; $w_{i} = \frac {1}{e(x_{i})}$ and the inverse of the probability of a mother being HIV-uninfected, conditional on covariates is given by $w_{i} = \frac {1}{1-e(x_{i})}$, where *e*(*x*_*i*_) is the propensity score. To estimate the HIV effect based on the weights, we defined the inverse probability weights with respect to HIV-infection status as;

2 $$\begin{array}{@{}rcl@{}} w_{i}=\frac{A_{i}}{e(x_{i})} + \frac{(1-A_{i})}{1-e(x_{i})}, A_{i} = \text{HIV status} =0, 1 \end{array} $$

The IPW was estimated as a weight on the PS using the p-scores obtained for mothers’ HIV status on confounders, place of residence, education, wealth quintile, mothers’ age, employment, whether a mother ever terminated a pregnancy, BMI, region, size of child at birth, BMI, marital status and maternal anaemia. To estimate the HIV effect on birth weight and on perinatal mortality using IPW, a weighted linear regression model and a weighted logistic regression model was fit using the inverse probability weights, respectively.

### Balance of measured covariates

#### Statistical comparison among the balancing methods

The balancing methods were assessed in their ability to balance the distribution of measured potentially confounded covariates between HIV-infected and HIV-uninfected mothers by reducing the standardised mean difference. The method that produced the smallest absolute standardised difference was then used to estimate the association of maternal HIV-infection on the outcomes. The standardised differences compared the prevalence [43] of the categorical covariates in HIV-infected and HIV-uninfected mothers, across the measured covariates. Even though there is no firm agreement on what value of the standardised difference denotes imbalance between exposed and unexposed subjects in the matched sample, the study adopted the decision criterion from some researchers who proposed that a standardised difference of 0.1 (10 per cent) denotes meaningful balance in the measured covariates [44].

In addition to balance checking using the standardised differences, we tested for balance by sub-classifying the propensity scores into quintiles. The full sample (HIV-infected mothers) was separated into five quintiles defined by their propensity scores. For the covariates, frequencies were compared before and after adjusting for propensity score quintile among HIV-infected and HIV-uninfected mothers. Specifically, the p-values for HIV status across the covariates were compared before and after adjustment for propensity score quintile to determine whether balance on the covariates was achieved.

### Moderator analysis

Moderation analysis has been used in various public health disciplines including studies on HIV [45, 46]. Most studies have examined the moderating effect of a variable by considering simple interaction effects [45, 47], while others have done moderation analysis using complex statistical methodologies such as the generalised estimating equations [46].

In a full moderation setting, one needs all information of maternal HIV-infection between different levels of ART status. However, in our study, we are limited in that ART status was available for only those mothers who were HIV-infected. Therefore, we did a partial moderation analysis that considered the association between ART status and birth weight and perinatal mortality among HIV-infected mothers on ART and HIV-infected mothers not on ART. We conducted an analysis of variance (ANOVA) to test the difference in mean birth weight between HIV-uninfected mothers, HIV-infected mothers on ART and HIV-infected mothers not on ART. The Bonferroni ad hoc test was used to measure the difference in means. We further tested the difference in proportion of perinatal mortality between HIV-uninfected mothers, HIV-infected mothers on ART and HIV-infected mothers not on ART. We used a logistic regression to assess the effect of ART on perinatal mortality as a moderating variable.

### Assumptions to validate HIV-infection effect

In order to identify the effect of HIV-infection on birth weight and perinatal mortality from cross-sectional data, several assumptions must be considered. The first two unverifiable assumptions are consistency and exchangeability (strongly ignorable assumption). The consistency assumption states that the observed outcome denoted as *Y*_*i*_ is equal to an individuals potential outcome *Y*_*i*_(*a*), *Y*_*i*_=*Y*_*i*_(*a*). The exchangeability assumption states that, conditional on measured covariates, the potential outcomes are independent of the exposure (*Y*(1),*Y*(0))∐*A*|*X*. This implies that once we control for the measured covariates, we reduce confounding. Therefore, we need to ensure that all covariates that affect maternal HIV status, birth weight and perinatal mortality are controlled for. The positivity assumption states that each subject must have a non-zero probability of being either HIV-infected or HIV-uninfected. An additional assumption is the Stable Unit Treatment Value Assumption (SUTVA) which assumes independence in the data between the different subjects. Since the design for the MDHS data follows a multi-stage cluster sampling, we assume there was no interdependence between clusters and because the subjects were randomly selected, hence, reducing selection bias.

### Sensitivity analysis

A sensitivity analysis was performed to measure the level of unobserved confounders that would affect the obtained estimates. The MDHS data did not include variables that were likely to influence birth weight and perinatal mortality. Variables such as, clinical factors, mothers diet, fetal height, were not available in the MDHS and were considered as unmeasured confounders. A sensitivity analysis method developed by Rosenbaum et al. [48] was used. The Hodges-Lehmann test and Wilcoxon Signed Rank test were used to conduct the sensitivity analysis. The maximum value of *Γ* was set to 1.3 with 0.05 increments. No unmeasured confounders implies that *Γ*=1. The rbounds package in R was used to obtain the upper and lower bounds on the *p*-value and point estimate [49].

## Results

A summary of the distribution of mothers’ baseline characteristics by mothers’ HIV status are shown in Table 1. Valid data was available for 4,111 and 4,759 under-five children whose mothers had an HIV test result, for the 2010 and 2015 MDHS, respectively. Analysis was done to test the effect of maternal HIV-infection on birth weight and perinatal mortality. From the 2010 data, 316 mothers were HIV-infected, 95 (49.5%) of them were on ART and 248 (79.5%) were residing in rural areas. Forty (12.7%) HIV-infected mothers were from the southern region, 179(56.7%) were aged 25 to 34 years and a third of them (n=103, 32.6%) were from poor households with 196(62.0%) having had a primary education. One hundred and twenty (38.7%) HIV-infected mothers had anaemia, 52(16.5%) reported to have ever terminated a pregnancy and 192(60.8%) were married. In 2015-16, 234(9.0%) of the mothers were HIV-infected of whom 168(71.8%) were from the rural area and 105(44.9%) were aged between 25 to 34 years old. Thirty nine (16.7%) of the HIV-infected mothers were in the middle class category, 144(61.5%) had a primary education and 167(71.4%) were married. One hundred and eighteen (50.4%) of the HIV-infected mothers had anaemia and 38(16.2%) reported to have ever terminated a pregnancy.

**Table 1:** Distribution of confounders between HIV-infected and HIV-uninfected mothers before matching for MDHS 2010 and 2015-16

**Characteristic**	**HIV status (2010)**			**HIV Status(2015)**		
	HIV-infected(%)	HIV-uninfected(%)	*p*-value	HIV-infected(%)	HIV-uninfected(%)	*p*-value
**Overall**	316(11.0)	2681(89.0)		234(9.0)	2456(91.0)	
**Residence**						
Urban	68(21.5)	320(11.9)		66(28.2)	409(16.7)	
Rural	248(78.5)	2361(88.1)	<0.001	168(71.8)	2047(83.4)	<0.001
**Region**						
Northern	40(12.7)	581(21.7)		30(12.8)	481(19.6)	
Central	76(24.1)	933(34.8)		49(20.9)	887(36.1)	
Southern	200(63.3)	1167(43.5)	<0.001	155(66.2)	1088(44.3)	<0.001
**Age**						
15-24	72(22.8)	983(36.7)		62(26.5)	1086(44.2)	
25-34	179(56.7)	1198(44.7)		105(44.9)	1025(41.7)	
35 above	65(20.6)	500(18.7)	<0.001	67(28.6)	345(14.1)	<0.001
**Wealth**						
Poor	103(32.6)	1004(37.5)		90(38.5)	1054(42.9)	
Middle	60(19.0)	609(22.7)		39(16.7)	478(19.5)	
Rich	153(48.4)	1068(39.8)	0.013	105(44.9)	924(37.6)	0.09
**Education**						
None	46(14.6)	322(12.0)		25(10.7)	240(9.8)	
Primary	196(62.0)	1829(68.2)		144(61.5)	1582(64.4)	
Secondary	70(22.2)	508(19.0)		62(26.5)	585(23.8)	
Tertiary	4(1.2)	22(0.8)	0.154	3(1.3)	49(2.0)	0.647
**Anaemia**						
No	190(61.3)	1939(74.2)		116(49.6)	1757(71.6)	
Yes	120(38.7)	675(25.8)	<0.001	118(50.4)	690(28.5)	<0.001
**Ever terminated pregnancy**						
No	264(83.5)	2376(88.6)		196(83.8)	2235(91)	
Yes	52(16.5)	304(11.3)	0.028	38(16.2)	221(9.0)	<0.001
**Marital Status**						
Never married	10(3.2)	78(2.9)		5(2.0)	119(4.9)	
Married	192(60.8)	2050(76.5)		167(71.4)	1928(78.5)	
Living together	30(9.5)	282(10.5)		13(5.6)	164(6.7)	
Widowed	20(6.3)	39(1.5)		10(4.3)	24(1.0)	
Divorced	39(12.3)	117(4.4)		18(7.7)	93(3.8)	
Not Living together	25(7.9)	115(4.3)	<0.001	21(9.0)	128(5.2)	<0.001
**Place of delivery**						
Home	13(4.4)	102(4.1)		5(2.3)	32(1.4)	
Government Hosp.	247(83.5)	2047(82.7)		200(90.9)	2051(88.4)	
Private	8(2.7)	71(2.9)		4(1.8)	58(2.5)	
Christian/Mission Hosp.	28(9.5)	255(10.3)	0.96	11(5.0)	180(7.8)	0.301
**Last birth C/section**						
No	294(93.0)	2520(94)		215(92.3)	3263(92.6)	
Yes	21(6.7)	158(5.9)	0.55	18(7.7)	180(7.4)	0.675

### Comparison before and after balancing confounders across HIV groups

Before matching, for both datasets, mothers’ demographic and health characteristics, region, place of residence, marital status, age of a mother, maternal anaemia, marital status and whether a mother ever terminated a pregnancy varied among HIV-infected and HIV-uninfected mothers as presented in Table 1. Wealth quintile was different among HIV-infected and HIV-uninfected mothers for the 2010 data only.

After applying the balancing methods on the covariates for the 2010 and 2015-16 datasets, no significant difference was observed for the covariates between the HIV-infected and HIV-uninfected, on the matched sample, as shown in Table 9 in the Appendix. Before balancing the differences, the absolute value of standardised difference was greater than 0.1 across the covariates. After balancing the covariates using the three methods, the absolute standardised difference for the three methods was less than 0.1 as shown in Tables 2 and 3. We further used the standardised differences obtained for the three methods to choose the balancing method that produced the best balance among the covariates and used it to estimate the effect of maternal HIV infection on birth weight and perinatal mortality. For both the 2010 and 2015-16 data, the IPTW gave smaller absolute standardised differences as compared to the PS match and the NN match method.

**Table 2:** Absolute standardised differences for before and after balancing the covariates with the three methods for the 2010 MDHS

**Covariates**	**Unmatched**	**NN Match**	**PS Match**	**IPW**
**Residence**	0.29	0.00	0.04	0.06
**Wealth**	0.13	0.05	0.03	0.00
**Age**	0.16	0.04	0.06	0.01
**Anaemia**	0.28	0.05	0.03	0.02
**Region**	0.37	0.11	0.03	0.03
**Education**	0.05	0.01	0.05	0.02
**Marital status**	0.02	0.03	0.02	0.00
**Size at birth**	0.05	0.02	0.03	0.04
**Ever terminated pregnancy**	0.15	0.01	0.1	0.04

**Table 3:** Absolute standardised differences for before and after balancing the covariates with the three methods for the 2015-16 MDHS

**Covariates**	**Unmatched**	**NN Match**	**PS Match**	**IPW**
**Residence**	0.30	0.01	0.02	0.01
**Wealth**	0.14	0.03	0.01	0.06
**Age**	0.39	0.10	0.03	0.06
**Anaemia**	0.43	0.02	0.06	0.01
**Region**	0.35	0.19	0.06	0.03
**Education**	0.02	0.01	0.06	0.01
**Marital status**	0.28	0.03	0.01	0.01
**Size at birth**	0.10	0.02	0.04	0.02
**Ever terminated pregnancy**	0.16	0.02	0.09	0.03

To further ensure that balance was obtained, we stratified the matched observations into quintiles according to their propensity scores. We observed that, within each quintile, there was no significant difference between HIV status for most of the covariates identified as confounders. These results are shown in Tables 10 and 11 in the Appendix. This demonstrated that balance was achieved.

### Maternal HIV effect estimation

Table 4 presents results for the effect of maternal HIV-infection on birth weight and perinatal mortality using the inverse probability weights. The results for the 2010 data showed that maternal HIV-infection was negatively associated with birth weight. Infants of HIV-infected mothers had a lower average birth weight of -25.3g (95% CI: -95.5, -7.4) as compared to infants of HIV-uninfected mothers. The 2015-16 results showed a significant positive association between maternal HIV infection status and birth weight. Infants of HIV-infected mothers had higher average birth weight 116.3g (95% CI: 27.8, 204.7) compared to infants of HIV-uninfected mothers. In addition, a comparison of the odds ratio of perinatal mortality between HIV-infected and HIV-uninfected mothers showed an increase in perinatal deaths in HIV-infected mothers in 2010. The odds of perinatal mortality was 1.5 times greater among infants of HIV-infected mothers compared to infants of HIV-uninfected mothers. However, in 2015, there was no significant difference in perinatal mortality between HIV-infected and HIV-uninfected mothers.

**Table 4:** The effect of maternal HIV-infection on birth weight and perinatal mortality

	**Child Health Outcomes 2010**		**Child Health Outcomes 2015-16**	
	Birth Weight	Perinatal Mortality	Birth Weight	Perinatal Mortality
	Mean.Diff(95% CI)	OR(95% CI)	Mean.Diff(95% CI)	OR(95% CI)
Unadjusted	-97.2(-160.4, -34)	1.8(1.2, 2.8)	52.6 (41.6, 125.5)	1.4(0.8, 2.6)
Adjusted weights	-25.3(-95.5, -7.4)	1.5(1.1, 3.1)	116.3(27.8, 204.7)	1.0(0.4, 1.6)

Variables age, residence, region, wealth, marital status, ever terminated a pregnancy, anaemia and body mass index were included in the propensity score model

Table 5 presents the effect of ART status on the association between maternal HIV-infection on birth weight and perinatal mortality among mothers who were HIV-infected. There was no difference in birth weight between infants born from HIV-infected mothers on ART and those born to HIV-infected mothers not on ART. However, a significant difference (*p*-value =0.02) in birth weight was observed between infants born to HIV-uninfected mothers and those born to HIV-infected mothers on ART as shown in Table 5. Infants of HIV-uninfected mothers had a higher average birth weight of 3231.3g (95% CI: 3208.8, 3253.9) as compared to the average birth weight of 3128.1g (95%: 2982.5, 3273.7) for infants born to HIV-infected mothers on ART.

**Table 5:** Mean Birth weight and proportion of perinatal deaths for HIV-uninfected and by ART status among HIV-infected mothers for the 2010 MDHS data

	**Birth weight**		**Perinatal Mortality**	
	**Mean (95% CI)**	***p****-value*	**Prop.(95% CI)**	***p****-value*
**HIV-infected on ART (*m***_**1**_**)**	3128.1(2982.5,3273.7)	0.64 (*m*_1_ vs *m*_2_)	4.3(0.2, 8.3)	0.03(*m*_1_ vs *m*_2_)
**HIV-infected not on ART(*m***_**2**_**)**	3031.9(2865.8, 3198.0)		9.4(3.5, 15.2)	
**HIV-uninfected** (*m*_3_)	3231.4(3208.8, 3253.9)	0.02(*m*_1_ vs *m*_3_)	4.0(3.4, 4.7)	0.9(*m*_1_ vs *m*_3_)

After observing a significant difference in prevalence of perinatal mortality between HIV-infected mothers on ART and those not on ART, we assessed the moderating effect of ART on the association between maternal HIV infection and perinatal mortality. There was a significant difference in perinatal mortality between HIV-infected mothers on ART and HIV-infected mothers not on ART as presented in Table 6. The odds of perinatal mortality was 3.9 times greater among infants of HIV-infected mothers not on ART as compared to infants of HIV-infected mothers on ART. In addition, there was no difference in perinatal mortality between HIV-infected mothers on ART and HIV-uninfected mothers.

**Table 6:** The effect of ART status on perinatal mortality

	**Perinatal Mortality**	
	**OR**	**95% CI**
**HIV-infected on ART**	∗∗	
**HIV-infected not on ART**	3.9	(1.1, 14.8)
**HIV-uninfected**	0.7	(0.2, 2.5)

∗∗ - reference category

Variables age, residence, region, wealth, marital status, ever terminated a pregnancy, anaemia and body mass index were included in the propensity score model

### Sensitivity analysis

A sensitivity analysis was done after matching to observe if the estimates obtained were sensitive to bias from unmeasured confounders. Table 12 in the Appendix presents results on the upper and lower bounds on the Wilcoxon Signed Rank *p*-value and the upper and lower bounds on the Hodges-Lehmann point estimate for birth weight. Based on the tables, the results for the sensitivity analysis showed that the *p*-value for the estimate of birth weight between HIV-infected and HIV-uninfected mothers with similarly measured covariates, would change with a small bias influenced by unmeasured confounders. The Hodges-Lehmann estimates similarly showed that a small bias influenced by unobserved differences would cause a change in the estimates.

## Discussion

The study set out to assess the effect of maternal HIV infection on birth weight and perinatal mortality and the effect of ART on these pregnancy outcomes. The study has shown that, in 2010, maternal HIV infection had an adverse effect on birth weight and perinatal mortality. However, in 2015-16, infants of HIV-infected mothers significantly weighed more than infants born to HIV-uninfected mothers. ART status had no effect on the association between maternal HIV infection and birth weight. However, ART did have a positive effect on the association between maternal HIV infection and perinatal mortality.

These findings from our study are similar to previous studies that reported a high risk of low birth weight among HIV-infected women [4, 9, 15, 50]. This has been attributed to the fact that HIV-infected women not on treatment have suppressed immune system which may have affected child growth and contributed to LBW among their infants [1, 4, 18]. Consistent with previous results [8, 18, 51], the study has also shown that perinatal mortality was higher among infants of HIV-infected mothers compared to those of HIV-uninfected mothers. These findings are in line with studies that have reported that LBW and perinatal mortality are inter-related and that LBW accounts for the majority of perinatal deaths [18, 51]. Since low birth weight was high among infants of HIV-infected mothers, we postulate that most of them did not survive after the first week from birth.

**Table 7:** Missing Pattern by studied sample

	**2010 MDHS data**		**2015 MDHS data**	
**Variable**	**Missing**	**Percent**	**Missing**	**Percent**
Birth weight	806	19.1	484	10.2
Terminated pregnancy	423	10.0	508	10.7
Residence	414	10.0	455	9.5
HIV-infection status	317	9.0	480	10.1
Size at birth	408	9.9	473	10.0
Anaemia	522	13.0	448	9.4
age	415	10.0	480	10.1
Marital status	415	10.0	463	9.7
perinatal deaths	434	10.6	739	15.0

There has been a lack of consensus on the effect of maternal HIV infection on birth weight with some studies attributing LBW to maternal HIV infection [4, 15, 17], while others attributing it to the use of ART [2, 8, 14, 16]. The findings from the current study, which suggest that the association between maternal HIV infection and birth weight was not affected by ART uptake, is consistent with findings of Xiao et al. [4] who found that ART exposure did not decrease or increase the risk of LBW in HIV-infected women. However, Gibango et al. [18] reported that HIV-positive mothers not on ART were likely to deliver an infant with low birth weight. Even though ART did not mitigate the association between maternal HIV infection and birth weight, it did mitigate the association between maternal HIV and perinatal mortality. Contrary to our findings, previous studies have shown that adverse pregnancy outcomes such as stillbirths are high among HIV-infected women on ART [52]. Lower perinatal deaths among HIV-infected mothers on ART may not entirely be as a result of the ART uptake but may also include uptake of good nutritional diet by the mothers. PMTCT programs through antenatal care (ANC) services ensure HIV-infected pregnant mothers are on ART and encourage good maternal nutrition and infant feeding practices. However, the 2010 MDHS report revealed that only 12% and 48% of pregnant women had their first ANC visit during the first trimester and during the fourth and fifth month of pregnancy, respectively [36]. PMTCT interventions through ANC clinics and community ART groups [53] may have been beneficial in improving perinatal mortality among infants of HIV-infected women but did not help in improving birth weight in 2010. Perhaps the benefits of the PMTCT programmes provided at ANC clinics during this period could have been more pronounced if frequency and timing of ANC visit was high.

In 2015-16, there was an increase in birth weight among infants born to HIV-infected mothers compared to infants born to HIV-uninfected mothers. This could be attributed to the fact that in July 2011, Malawi was one of the first countries to implement Option B+ approach as an effort to deal with HIV/AIDS and its impact. This is an intervention where all pregnant women living with HIV were offered ART for life irrespective of their CD4 count [54]. Reports show that with the introduction of option B+, there was a huge improvement in PMTCT in Malawi [55]. The estimated percentage of pregnant women living with HIV who received ART drugs for PMTCT in Malawi was 84% in 2016 [56]. Perhaps the HIV-infected women were part of this intervention and it may have helped them boost their immunity [16, 55] which in turn resulted in good child growth. The findings of the current study further showed that perinatal mortality was similar between HIV-infected and HIV-uninfected. The results may suggest that since infants of HIV-infected mothers had higher birth weight then they were less likely to die of LBW or prematurity. In addition, through antenatal care services, HIV-infected pregnant mothers are put on ART and are encouraged to attend at least four ANC visits where they receive the required iron supplements, folic acid and Ready to Use Therapeutic Food (RUTF) [57]. This may have helped HIV-infected pregnant mothers to maintain a balanced nutritional status which in turn helped in child growth as compared to HIV-uninfected mothers who only receive iron supplements and folic acids without additional food supplements. Previous studies have also shown that prenatal and antenatal care visits among pregnant mothers result in a decrease in perinatal mortality [58, 59]. The findings from our study may suggest a major improvement in the implementation of PMTCT interventions by the Ministry of Health and other key stakeholders.

**Table 8:** Confounder identification

**Variable**	**Outcome**	**Exposure**	
	Birth weight (*p*-value)	Perinatal death(*p*-value)	HIV status (*p*-value)
Age of a mother	<0.001	<0.001	<0.001
Region	NA	0.006	<0.001
Residence	0.016	0.946	<0.001
Education	0.011	0.43	0.012
Size of child at birth	<0.001	0.06	0.31
Ever had terminated pregnancy	0.845	<0.001	0.007
Anaemia	<0.001	0.90	<0.001
Marital status	NA	NA	<0.001
place of delivery	NA	0.249	0.97
Wealth	0.97	0.286	0.008
(a) Confounders that influence HIV and the outcomes for the 2010 MDHS			
	Birth weight (*p*-value)	Perinatal death(*p*-value)	HIV status (*p*-value)
Age of a mother	<0.001	0.226	<0.001
Region	0.133	0.35	<0.001
Residence	0.49	0.126	<0.001
Education	0.096	0.716	<0.001
BMI	0.017	NA	0.577
Size of child at birth	<0.001	<0.001	0.304
Ever had terminated pregnancy	0.730	<0.001	0.001
Anaemia	0.21	0.50	<0.001
Marital status	NA	NA	<0.001
Place of delivery	NA	0.26	0.41
Wealth	0.001	0.287	0.017
(b) Confounders that influence HIV and the outcomes for the 2015-16 MDHS			

*Confounder Identification* A verification of the variables was done by fitting a linear regression model for the effect of each confounder on child birth weight and did a crosstabulation using Pearsons Chi-square test for the effect of the confounders on perinatal mortality. We also checked the association between the identified variables and mothers HIV-infection status. Variables that were not known to influence either HIV status or birth weight or perinatal death were labelled as “not applicable”

**Table 9:** Distribution of Confounders between HIV-infected and HIV-uninfected after matching for MDHS 2010 and 2015-16 data

**Characteristic**	**Mothers HIV status (2010)**			**Mothers HIV Status(2015)**		
	HIV-infected	HIV-uninfected	*p*-value	HIV-infected(%)	HIV-uninfected(%)	*p*-value
**Overall**	301(51.2)	306(48.8)		387(50.1)	386(49.9)	
**Residence**						
Urban	66(20.6)	58(19.0)		112(28.9)	120(31.1)	
Rural	255(79.4)	248(81)	0.61	275(71.1)	266(68.9)	0.52
**Region**						
Northern	42(13.1)	37(12.1)		46(11.9)	41(10.6)	
Central	84(26.2)	101(33.0)		91(23.5)	117(30.3)	
Southern	195(60.8)	168(54.9)	0.17	250(64.6)	228(59.1)	0.10
**Age**						
15-24	78(24.3)	97(31.7)		93(24.0)	90(23.3)	
25-34	173(53.9)	122(39.9)		181(46.8)	197(51.0)	
35 above	70(21.8)	87(28.4)	0.13	113(29.2)	99(25.7)	0.44
**Wealth**						
Poor	100(31.2)	96(31.4)		144(37.2)	137(35.5)	
Middle	70(21.8)	65(21.2)		65(16.8)	67(17.4)	
Rich	151(47.0)	145(47.4)	0.98	178(46)	182(47.1)	0.88
**Education**						
None	50(15.6)	41(13.4)		44(11.4)	54(14.0)	
Primary	199(62)	206(67.3)		242(62.5)	213(55.2)	
Secondary	69(21.5)	57(18.6)		94(24.3)	107(27.7)	
Tertiary	3(0.9)	2(0.6)	0.57	7(1.8)	12(3.1)	0.17
**Anaemia**						
No	193(60.1)	196(64.1)		196(50.7)	193(50.0)	
Yes	128(39.9)	116(35.9)	0.31	191(49.3)	193(50.0)	0.86
**Ever terminated pregnancy**						
No	272(84.7)	259(84.6)		332(85.8)	322(83.4)	
Yes	49(15.3)	47(15.4)	0.97	55(14.2)	64(16.6)	0.30
**Marital Status**						
Never married	8(2.5)	3(1.0)		10(2.6)	8(2.1)	
Married	197(61.4)	202(66.0)		273(70.5)	275(71.2)	
Living together	32(10.0)	36(11.8)		18(4.7)	26(6.7)	
Widowed	21(6.5)	5(1.6)		13(3.4)	12(3.1)	
Divorced	37(11.5)	22(7.2)		37(9.6)	23(6.0)	
Not Living together	26(8.1)	38(12.4)	0.03	36(9.3)	42(10.9)	0.36

**Table 10:** Comparison of Covariates for HIV status Before and After Propensity Score Stratification

**Covariate**	***p****-value(2010))*		***p****-value(2015-16)*	
	**Before stratification**	**After stratification**	**Before stratification**	**After Stratification**
**Age**				
15-24	<0.001	0.800	<0.001	0.767
25-34	0.009	0.922	0.567	0.316
35 above	0.02	0.893	<0.001	0.14
**Wealth**				
Poor	0.553	0.440	0.04	0.78
Medium	0.02	0.817	0.34	0.70
Rich	0.01	0.618	0.004	0.56
**Residence**				
Urban	<0.001	0.424	<0.001	0.25
Rural	<0.001	0.424	<0.001	0.25
**Region**				
Northern	0.001	0.514	<0.001	0.08
Central	0.001	0.681	<0.001	0.649
Southern	<0.001	0.368	<0.001	0.5
**Anemia**				
No	0.04	0.537	<0.001	0.21
Yes	0.04	0.537	<0.001	0.21

**Table 11:** Quintiles Based on estimated Propensity Scores

**Distribution**	**Sample (N)**		***P****-value*				
	**HIV-uninfected**	**HIV-infected**	**age**	**Residence**	**Region**	**Anaemia**	**Ever Terminated pregnancy**
(a) HIV status Group Differences on Covariates age, residence, region, anemia and ever terminated pregnancy, by Strata Based on Propensity Score							
Quintiles on the matched sample for 2010 MDHS							
Quintile 1	719	23	0.06	0.069	0.688	0.41	0.02
Quintile 2	692	51	0.502	0.653	0.513	0.1	0.1
Quintile 3	683	58	0.347	0.211	0.06	0.95	0.59
Quintile 4	656	86	0.9	0.1	0.39	0.1	0.42
Quintile 5	590	152	0.5	0.97	0.3	0.72	0.22
(b) HIV status Group Differences on Covariates age, residence, region, anemia and ever terminated pregnancy, by Strata Based on Propensity Score							
Quintiles on the matched sample for 2015-16 MDHS							
Quintile 1	870	24	0.236	0.04	0.67	0.34	0.83
Quintile 2	855	39	0.38	1.00	0.323	0.16	0.37
Quintile 3	821	72	0.06	0.29	0.07	0.07	0.87
Quintile 4	796	98	0.12	0.37	0.04	0.16	0.39
Quintile 5	713	180	0.04	0.903	0.07	0.526	0.19

### Strengths and limitations

One of the strengths of the study is that data originating from a large survey was used and hence, using a rich data with a vast number of socio-demographic characteristics of participants. The study also used matching and weighting methods to reduce confounding for the observed covariates. Several limitations of the study must be addressed. The study used survey data that collected retrospective information on births and deaths. Under-reporting of births and deaths for children who were not living at the time of the survey may be common. Mothers may have been reluctant to talk about their dead children either because the subject brought back sad memories or because their culture discouraged mentioning of the dead. Coupled to that, the analysis was done on cross-sectional data and despite controlling for confounding, it was difficult to assess the causal effect of maternal HIV status on birth weight and on perinatal and infant mortality, as the data is very likely to have unmeasured covariates that may act as confounders. In addition, it is difficult to measure the change in birth weight among HIV-infected mothers over a certain period of time. Furthermore, some important confounding risk factors that are known to affect birth weight were not available in the data. These include; unfavourable genetic factors which predispose infants to be smaller in size and length [26], maternal nutrition, maternal passive smoking, drinking, folic acid supplement use, taking drugs and family history of birth defects [50]. Therefore, the obtained estimates may be sensitive to unmeasured confounders. In addition, knowledge of ART timing, initiation of ART and type of ART regimen were not available in the data. These are known to have influenced the risk of adverse birth outcomes in HIV-infected mothers. On the other hand, knowledge on date of HIV acquisition was not available in the data hence it may be possible that a mother was infected with HIV after infant birth. In this case, HIV status would not affect birth weight. Furthermore, ART status was not available within the 2015-16 MDHS data. Hence, conclusions about the effect of ART status on birth weight can only be attributed to the 2010 data and may not explain changes in birth weight for the 2015-16 data. The assumptions stated to estimate the maternal HIV effect on the outcomes were not tested, hence estimates may be prone to bias.

**Table 12:** Rosenbaum’s Sensitivity Analysis

	***P****-value*		**Estimate**	
**Gamma**	**Lower bound**	**Upper bound**	**Lower**	**Upper**
(a) Wilcoxon Signed Rank P-Value and Hodges-Lehmann Point Estimate for 2010 MDHS				
1	0.001	0.00	400	400
1.1	0.0002	0.00	350	450
1.2	0.0001	0.01	300	500
1.3	<0.001	0.01	300	500
1.4	<0.001	0.02	250	550
1.5	<0.001	0.03	250	550
(b) Wilcoxon Signed Rank P-Value and Hodges-Lehmann Point Estimate for 2015-16 MDHS				
1	0.0004	0.00	-83.3	-83.3
1.1	0.0001	0.00	-133.3	-133.3
1.2	<0.001	0.01	-150.1	150.1
1.3	<0.001	0.01	-166.7	166.7
1.4	<0.001	0.03	-183.3	183.3
1.5	<0.001	0.06	-200	200

## Conclusion

Our findings suggest that maternal HIV infection had an impact on birth weight and perinatal mortality. The uptake of ART had a moderating effect on the association between maternal HIV-infection and perinatal mortality with higher rates among HIV-infected mothers not on ART. The study has also shown a significant increase in birth weight between the 2010 and 2015-16 MDHS. In addition, the findings show a change in effect of maternal HIV-infection on perinatal deaths between the period 2010 and 2015. These results may be explained by the recent success of policies and interventions under the PMTCT programme such as wide coverage of antenatal care services and the option B+ strategy. Therefore, there is a need to emphasise the importance of uptake of PMTCT guidelines for effective child health outcomes. Statistical methods that control for confounders are useful tools to address the lack of randomised control trials in assessing the effect of maternal HIV on pregnancy outcomes based on observational data. We recommend future studies to consider using causal inference techniques when an investigation of association of risk factor and outcome is undertaken using observational studies such as health surveys.

## Appendix

## Data Availability

The data that support the findings of this study are available upon request from the Demographic and Health Survey (DHS) website. Upon approval, full access is granted to all unrestricted survey datasets.

## Abbreviations

ANC: Antenatal clinic
ANOVA: Analysis of variance
ART: Anti-retroviral therapy
HIV: Human immune-deficiency virus
IPW: Inverse probability weights
LBW: Low birth weight
LMIC: Low-middle income countries
Malawi Demographic and health survey; MTCT: Mother to child transmission
NN: Nearest neighbour
PMTCT: Prevention of mother to child transmission
PS: Propensity score
Randomised control trial; SUTVA: Stable unit treatment value assumption
WHO: World health organisation

## References

[1] Dreyfuss ML, Msamanga GI, Spiegelman D, Hunter DJ, Urassa EJ, Hertzmark E, Fawzi WW (2001). Determinants of low birth weight among HIV-infected pregnant women in Tanzania. Am J Clin Nutr.

[2] Rollins NC, Coovadia HM, Bland RM, Coutsoudis A, Bennish ML, Patel D, Newell M-L (2007). Pregnancy outcomes in hiv-infected and uninfected women in rural and urban south africa. JAIDS J Acquir Immune Defic Syndr.

[3] Claeson M, Gillespie D, Mshinda H, Troedsson H, VC (2003). Bellagio study group on child survival: Knowledge into action for child survival. Lancet.

[4] Xiao P-L, Zhou Y-B, Chen Y, Yang M-X, Song X-X, Shi Y, Jiang Q-W (2015). Association between maternal HIV infection and low birth weight and prematurity: a meta-analysis of cohort studies. BMC Pregnancy Childbirth.

[5] Ellis J, Williams H, Graves W, Lindsay MK (2002). Human immunodeficiency virus infection is a risk factor for adverse perinatal outcome. Am J Obstet Gynecol.

[6] Brocklehurst P, French R (1998). The association between maternal hiv infection and perinatal outcome: a systematic review of the literature and meta-analysis. BJOG: Int J Obstet Gynecol.

[7] Ticconi C, Mapfumo M, Dorrucci M, Naha N, Tarira E, Pietropolli A, Rezza G (2003). Effect of maternal hiv and malaria infection on pregnancy and perinatal outcome in Zimbabwe. J Acquir Immune Defic Syndr 1999.

[8] Bayou G, Berhan Y. Perinatal mortality and associated risk factors: a case control study. Ethiop J Health Sci. 2012; 22(3). PMC3511893 23209349

[9] Nazli A, Chan O, Dobson-Belaire WN, Ouellet M, Tremblay MJ, Gray-Owen SD, Arsenault AL, Kaushic C (2010). Exposure to hiv-1 directly impairs mucosal epithelial barrier integrity allowing microbial translocation. PLoS Pathog.

[10] Nkhoma ET, Kalilani-Phiri L, Mwapasa V, Rogerson SJ, Meshnick SR (2012). Effect of hiv infection and plasmodium falciparum parasitemia on pregnancy outcomes in Malawi. Am J Trop Med Hyg.

[11] World Health Organization, et al.UNICEF-WHO low birthweight estimates: levels and trends 2000–2015. No. WHO/NMH/NHD/19.21. United Nations Children’s Fund (UNICEF). 2019.

[12] Oza S., Lawn J. E., Hogan D. R., Mathers C., Cousens S. N. (2014). Neonatal cause-of-death estimates for the early and late neonatal periods for 194 countries: 2000–2013. Bull World Health Organ.

[13] Joint United Nations Programme on HIV/AIDS (2013). Progress report on the global plan towards the elimination of new HIV infections among children by 2015 and keeping their mothers alive.

[14] Cotter AM, Garcia AG, Duthely ML, Luke B, O’Sullivan MJ (2006). Is antiretroviral therapy during pregnancy associated with an increased risk of preterm delivery, low birth weight, or stillbirth?. J Infect Dis.

[15] Szyld EG, Warley EM, Freimanis L, Gonin R, Cahn PE, Calvet GA, Duarte G, Melo VH, Read JS, Group NPS (2006). Maternal antiretroviral drugs during pregnancy and infant low birth weight and preterm birth. Aids.

[16] Marazzi MC, Palombi L, Nielsen-Saines K, Haswell J, Zimba I, Magid NA, Buonomo E, Scarcella P, Ceffa S, Paturzo G (2011). Extended antenatal use of triple antiretroviral therapy for prevention of mother-to-child transmission of hiv-1 correlates with favorable pregnancy outcomes. Aids.

[17] Tuomala RE, Watts DH, Li D, Vajaranant M, Pitt J, Hammill H, Landesman S, Zorrilla C, Thompson B (2005). Improved obstetric outcomes and few maternal toxicities are associated with antiretroviral therapy, including highly active antiretroviral therapy during pregnancy. JAIDS J Acquir Immune Defic Syndr.

[18] Gibango N, Mda S, Ntuli T (2018). Factors associated with delivering premature and/or low birth weight infants among pregnant hiv-positive women on antiretroviral treatment at dr george mukhari hospital, south africa. South Afr J Infect Dis.

[19] Chen JY, Ribaudo HJ, Souda S, Parekh N, Ogwu A, Lockman S, Powis K, Dryden-Peterson S, Creek T, Jimbo W (2012). Highly active antiretroviral therapy and adverse birth outcomes among hiv-infected women in botswana. J Infect Dis.

[20] Bian Y, Zhang Z, Liu Q, Wu D, Wang S (2013). Maternal risk factors for low birth weight for term births in a developed region in china: a hospital-based study of 55,633 pregnancies. J Biomed Res.

[21] Khan M, Arbab M, Murad M, Khan M, Abdullah S (2014). Study of factors affecting and causing low birth weight. J Sci Res.

[22] Nascimento LFC, Moreira DA (2009). Are environmental pollutants risk factors for low birth weight?. Cad Saúde Pública.

[23] Gebremedhin M, Ambaw F, Admassu E, Berhane H (2015). Maternal associated factors of low birth weight: a hospital based cross-sectional mixed study in tigray, northern ethiopia. BMC Pregnancy Childbirth.

[24] Ngwira A, Stanley CC (2015). Determinants of low birth weight in malawi: Bayesian geo-additive modelling. PloS One.

[25] Metgud CS, Naik VA, Mallapur MD (2012). Factors affecting birth weight of a newborn–a community based study in rural karnataka, india. PloS One.

[26] Dubois L, Kyvik KO, Girard M, Tatone-Tokuda F, Pérusse D, Hjelmborg J, Skytthe A, Rasmussen F, Wright MJ, Lichtenstein P (2012). Genetic and environmental contributions to weight, height, and bmi from birth to 19 years of age: an international study of over 12,000 twin pairs. PLOS One.

[27] Rosenbaum PR, Rubin DB (1983). The central role of the propensity score in observational studies for causal effects. Biometrika.

[28] Lunceford JK, Davidian M (2004). Stratification and weighting via the propensity score in estimation of causal treatment effects: a comparative study. Stat Med.

[29] Cole SR, Hernan MA (2008). Constructing inverse probability weights for marginal structural models. Am J Epidemiol.

[30] Austin PC (2011). An Introduction to Propensity Score Methods for Reducing the Effect of Confounding in Observational. Multivar Behav Res.

[31] Rosenbaum PR, Rubin DB (1985). Constructing a control group using multivariate matched sampling methods that incorporate the propensity score. Am Stat.

[32] He H, Hu J, He J. Overview of propensity score methods. In: Statistical Causal Inferences and Their Applications in Public Health Research. Springer International Publishing: 2016. p. 29–48.

[33] Sainani KL (2012). Propensity scores: uses and limitations. PM&R.

[34] Austin PC, Mamdani MM (2006). A comparison of propensity score methods: a case-study estimating the effectiveness of post-ami statin use. Stat Med.

[35] d’Agostino RB (1998). Tutorial in biostatistics: propensity score methods for bias reduction in the comparison of a treatment to a non-randomized control group. Stat Med.

[36] National Statistical Office (NSO) and ICF Macro (2011). Malawi Demographic and Health Survey 2010.

[37] National Statistical Office (NSO) [Malawi] and ICF (2017). Malawi Demographic and Health Survey 2015–16.

[38] Austin PC (2011). An introduction to propensity score methods for reducing the effects of confounding in observational studies. Multivar Behav Res.

[39] Rubin DB (1973). Matching to remove bias in observational studies. Biometrics.

[40] Stuart EA (2010). Matching methods for causal inference: A review and a look forward. Stat Sci Rev J Inst Math Stat.

[41] Rosenbaum PR, Rubin DB (1984). Reducing bias in observational studies using subclassification on the propensity score. J Am Stat Assoc.

[42] Austin PC (2011). Optimal caliper widths for propensity-score matching when estimating differences in means and differences in proportions in observational studies. Pharm Stat.

[43] Austin PC (2009). Balance diagnostics for comparing the distribution of baseline covariates between treatment groups in propensity-score matched samples. Stat Med.

[44] Normand S-LT, Landrum MB, Guadagnoli E, Ayanian JZ, Ryan TJ, Cleary PD, McNeil BJ (2001). Validating recommendations for coronary angiography following acute myocardial infarction in the elderly: a matched analysis using propensity scores. J Clin Epidemiol.

[45] Kim SC, Messing S, Shah K, Luque AE (2013). Effect of highly active antiretroviral therapy (haart) and menopause on risk of progression of cervical dysplasia in human immune-deficiency virus-(hiv-) infected women. Infect Dis Obstet Gynecol.

[46] Fabian KE, Huh D, Kemp CG, Nevin PE, Simoni JM, Andrasik M, Turan JM, Cohn SE, Mugavero MJ, Rao D. Moderating factors in an anti-stigma intervention for african american women with hiv in the united states: A secondary analysis of the unity trial. AIDS Behav. 2019:1–11. 10.1007/s10461-019-02557-x PMC6860974 31218545

[47] Baron RM, Kenny DA (1986). The moderator–mediator variable distinction in social psychological research: Conceptual, strategic, and statistical considerations. J Pers Soc Psychol.

[48] Rosenbaum PR. Observational studies. In: Observational Studies. Springer-Verlag New York: 2002. p. 1–17.

[49] Rosenbaum PR. Sensitivity Analysis in Observational Studies In: Everitt BS, Howell DC, editors. Encyclopedia of Statistics in Behavioral Science, vol 4: 2005. p. 1809–14.

[50] Guo L, Qu P, Zhang R, Zhao D, Wang H, Liu R, Mi B, Yan H, Dang S (2018). Propensity score-matched analysis on the association between pregnancy infections and adverse birth outcomes in rural northwestern china. Sci Rep.

[51] Naniche D, Bardají A, Lahuerta M, Berenguera A, Mandomando I, Sanz S, Aponte JJ, Sigauque B, Alonso PL, Menéndez C (2009). Impact of maternal human immunodeficiency virus infection on birth outcomes and infant survival in rural mozambique. Am J Trop Med Hyg.

[52] Stringer EM, Kendall MA, Lockman S, Campbell TB, Nielsen-Saines K, Sawe F, Cu-uvin S, Wu X, Currier JS. Pregnancy outcomes among hiv-infected women who conceived on antiretroviral therapy. PloS one. 2018; 13(7). 10.1371/journal.pone.0199555 PMC6051581 30020964

[53] van Lettow M, Landes M, van Oosterhout J, Schouten E, Phiri H, Nkhoma E, Kalua T, Gupta S, Wadonda N, Jahn A (2018). Prevention of mother-to-child transmission of hiv: a cross-sectional study in malawi. Bull World Health Organ.

[54] M.N.A.C.Malawi aids response progress report 2015. 2015.

[55] Chimbwandira F, Mhango E, Makombe S, Midiani D, Mwansambo C, Njala J, Chirwa Z, Jahn A, Schouten E, Phelps BR (2013). Impact of an innovative approach to prevent mother-to-child transmission of hiv—malawi, july 2011–september 2012. MMWR Morb Mortal Wkly Rep.

[56] Jahn A, Harries AD, Schouten EJ, Libamba E, Ford N, Maher D, Chimbwandira F (2016). Scaling-up antiretroviral therapy in malawi. Bull World Health Organ.

[57] Corbett M (2005). Support for plwhas in malawi. Field Exchange 25.

[58] Chihana ML, Price A, Floyd S, Mboma S, Mvula H, Branson K, Saul J, Zaba B, French N, Crampin AC (2015). Maternal hiv status associated with under-five mortality in rural northern malawi: a prospective cohort study. J Acquir Immune Defic Syndr 1999.

[59] Dowswell T, Carroli G, Duley L, Gates S, Gülmezoglu AM, Khan-Neelofur D, Piaggio G. Alternative versus standard packages of antenatal care for low-risk pregnancy. Cochrane Database Syst Rev. 2015;7. 10.1002/14651858.CD000934.pub3 PMC7061257 26184394

